# Opportunities for improvement in nursing homes: Variance of six patient safety climate factor scores across nursing homes and wards—Assessed by the Safety Attitudes Questionnaire

**DOI:** 10.1371/journal.pone.0218244

**Published:** 2019-06-19

**Authors:** Ellen Catharina Tveter Deilkås, Dag Hofoss, Bettina S. Husebo, Gunnar Tschudi Bondevik

**Affiliations:** 1 The Norwegian Directorate of Health, Oslo, Norway; 2 Health Services Research Unit, Akershus University Hospital, Lørenskog, Norway; 3 Lovisenberg Diaconal University College, Oslo, Norway; 4 Centre for Elderly and Nursing Home Medicine, Department of Global Public Health and Primary Care, University of Bergen, Bergen, Norway; 5 Municipality of Bergen, Bergen, Norway; 6 Section for General Practice, Department of Global Public Health and Primary Care, University of Bergen, Bergen, Norway; 7 National Centre for Emergency Primary Health Care, NORCE Norwegian Research Centre, Bergen, Norway; Indiana University, UNITED STATES

## Abstract

**Introduction:**

Safety climates are perceptions of safety culture shared by staff in organizational units. Measuring staff perceptions of patient safety culture by using safety climate surveys is a possible way of addressing patient safety. Studies have documented that patient safety climates vary significantly between work sites in hospitals. Across-ward variations in the measurements of safety climate factor scores may indicate ward-specific risk of adverse events related to patient care routines, work environment, staff behaviour, and patient results. Variation in patient safety climates has not yet been explored in nursing homes.

**Objectives:**

To investigate whether the Norwegian translation of the Safety Attitudes Questionnaire—Ambulatory Version is useful to identify significant variation in the patient safety climate factor scores: Teamwork climate, Safety climate, Job satisfaction, Working conditions, Stress recognition, and Perceptions of management, across wards in nursing homes.

**Methods:**

Four hundred and sixty three employees from 34 wards in five nursing homes were invited to participate. Cronbach alphas were computed based on individual respondents’ scores on the six patient safety climate factor scores. Intraclass correlation coefficients were calculated by multilevel analysis to measure patient safety climate variance at ward level.

**Results:**

Two hundred and eighty eight (62.2%) returned the questionnaire. At ward level Intraclass correlation coefficients (ICCs) for the factors were 10.2% or higher for the factors Safety climate, Working conditions and Perceptions of management, 2.4% or lower for Teamwork climate, Job satisfaction, and zero for Stress recognition. ICC for variance at nursing home level was zero or less than one per cent for all factor scores.

**Conclusions:**

Staff perceptions of Safety climate, Working conditions and Perceptions of management varied significantly across wards. These factor scores may, therefore, be used to identify wards in nursing homes with high and low risk of adverse events, and guide improvement resources to where they are most needed.

## Introduction

Patient safety improvement seems to be related to healthcare organizations’ ability to address and improve safety culture [1, 2]. Patient safety climate survey scores may show variation in staff perceptions across organizational units [3]. Such variation may indicate risks related to leadership or other aspects of organization. That offers the opportunity to address patient safety improvement through organizational interventions, e.g. mentoring leaders to conduct patient safety walkrounds, or facilitate improvement board meetings and informal regular meetings where frontline staff may plan and evaluate implementation of improvement efforts [4, 5]. Patient safety culture involves leader and staff interactions, attitudes, routines, awareness, and practices that influence risks of adverse events in patient care [6]. A cultural trait may for example be how leaders facilitate dialogue with staff to uncover negative relationships and behaviour, promote mutual understanding of the causes of adverse events, and establish consensus regarding safety priorities [7].

Variation in safety culture in hospitals has been associated with variation in rates of adverse events [8–10]. Studies have also found variations in patient safety culture in nursing homes, and associations with clinical outcomes, like the prevalence of patients injured by falls [11, 12].

Some studies have shown that safety cultures are less developed in nursing homes than in hospitals–which is a rather unpleasant finding, given that adverse event rates increase with patient age [13–15]: nursing home patients are at high risk of adverse events due to their age, non-specific presentation of illnesses, cognitive impairment, complex multiple-disease conditions, polypharmaceutic errors and drug interactions.

Efforts to address patient safety culture in healthcare are widespread [16, 17]. Measuring staff perceptions of patient safety culture with safety climate surveys is a relevant approach. Safety climate is the measurement of perceptions of safety culture shared amongst staff in an organizational unit. Although staff perceptions of safety culture are influenced by their profession or role, and facility characteristics such as ownership [18], valid safety climate measurements can identify levels of staff perceptions, the extent to which they are shared, and how they vary across organizational units. Such assessments may predict variability in risk related to tasks, work environment, staff behaviour, and patient outcome [19, 20]. They give leaders the opportunity to address cultural obstacles. One example may be that staff in some units feel that it is difficult to speak up about risks and short-cuts in patient care. Climate measurements will indicate such problems, which could be further specified by dialogue with staff during a patient safety walkround. Patient safety walkrounds are conducted by a senior leader or executive, and set up to deepen clinical staff and leaders understanding of risks in patient care at their own ward. Improvement opportunities could be addressed in the walkround by discussing what the safety standards should be, setting ward-specific quality targets and facilitating improvement board meetings. Additional improvement could be provided by establishing multidisciplinary quality teams to work continually with improvement [6, 21–23]. Modak et al developed the Ambulatory Version of the Safety Attitudes Questionnaire (SAQ-A) to measure patient safety climates in an outpatient setting [24]. The original version included six patient safety climate factors: Teamwork climate, Safety climate, Job satisfaction, Working conditions, Stress recognition, and Perceptions of management. In the first report from our study, we confirmed that this six factor model could be identified in Norwegian nursing homes [25]. In the second paper we documented factor score variations by age, gender, position, profession and mother tongue [26]. The aim of this third paper is to explore the degree to which the Norwegian version of the SAQ-A identifies variation in safety climate perceptions across wards in Norwegian nursing homes. No clustering at ward level would imply that the entire variance in patient safety climate scores was across individual responders, and that, accordingly, no ward was a more promising candidate for patient safety improvement intervention than any other. Considerable variation across nursing home wards would imply that the SAQ-A is useful for identifying nursing home wards with high and low scores, and to steer patient safety improvement work towards work places with lower scores on specific patient safety climate factors.

## Material and methods

### Sample

This is an observational study with a cross-sectional design. The study was conducted in all five nursing homes in Tønsberg, which is an average-sized Norwegian town and municipality with 42,000 inhabitants. Tønsberg was chosen as it was the first municipality in Norway to pilot the concept “Patient and user safe municipalities” for Norway’s national patient safety program. Recruiting the nursing homes for this study was part of the national pilot role for the municipality. The number of patients in each nursing home varied between 38 and 101. In total, there were 366 patients. In these five nursing homes 765 employees were nested in 34 wards. In our analysis, we did not include health care providers who worked less than/equal to 20% of a full-time position or were on leave during the study period (n = 302). In the latter group, most employees worked only one weekend every third or fourth week. Most of the remaining 463 employees were registered nurses or nursing assistants.

### Data collection

#### Survey

The original version of the SAQ-A questionnaire was translated from English to Norwegian following modified principles adapted from Beaton et al [27]. Based on a back-translated version, an expert committee made adjustments to avoid misunderstandings and adapt the questionnaire to the Norwegian nursing home setting. For instance, the original SAQ-A statement “Medical errors are handled appropriately in this office” was changed to “Medical errors are handled appropriately in this nursing home ward”, and “Nurse input is well received in this office” was changed to “Staff input is well received in this nursing home ward”. The pre-final version was evaluated by a group of health care providers in nursing homes. Based on their feedback, the final version of the Norwegian SAQ-A questionnaire for nursing homes was developed.

Data were collected in February 2016. Information about the study was presented on posters in the nursing home wards and in handouts to all participants prior to–and during–the data collection. Key administrative persons in the nursing homes distributed a paper version of the SAQ-A to the employees, and reminded them one week before deadline. It took approximately 15 minutes to complete the SAQ-A. We did include part-time workers, however, as this was a study on safety climate amongst employees, we excluded those working in very small positions ≤ 20%. To ensure confidentiality, filled-in questionnaires were returned anonymously in boxes placed in the nursing home wards. Questionnaires were scanned into an SPSS data file for analysis.

To protect the confidentiality of the respondents, feedback reports were only produced for wards with five respondents or more.

#### Variables, scores and measurements

The SAQ-A is a 62-item questionnaire where respondents rate their agreement using a five-point Likert scale. Before analysis, scores of negatively worded items were reversed, so that higher scores in the data set always indicate a more positive evaluation of the unit’s patient safety climate. Table 1 presents 28 of 62 items of the SAQ-A for nursing homes, which corresponds to the measurement model of SAQ which has been tested and validated in a previous study [25]. Items in the SAQ-A not covered by the six factors in the original model were kept in the questionnaire because they considered useful for local improvement processes and discussions. Factor scores for each individual respondent were computed as (mean value of item scores that belong to the factor—1) *25, so that the score”1” is transformed to”0”,”2” to”25”,”3” to”50”,”4” to”75”, and”5” to”100”.

**Table 1:** The six patient safety climate factors and corresponding items in the validated Norwegian translation of the Safety Attitudes Questionnaire–Ambulatory Version (SAQ-A) for nursing homes.

**Teamwork climate** Cronbach’s alpha: 0.655
Input from personnel is well received in this nursing home ward.
In this nursing home ward, it is difficult to speak up if I perceive a problem with patient care.*
Disagreements in this nursing home ward are resolved appropriately (i.e., not who is right but what is best for the patient).
I have the support I need from other personnel to care for patients.
**Safety climate** Cronbach’s alpha: 0.738
I would feel safe being treated here as a patient.
Medical errors are handled appropriately in this nursing home ward.
I receive appropriate feedback about my performance.
In this nursing home ward, it is difficult to discuss errors.*
I am encouraged by my colleagues to report any patient safety concerns I may have.
The culture in this nursing home ward makes it easy to learn from the errors of others.
I know the proper channels to direct questions regarding patient safety in this nursing home ward.
**Job satisfaction** Cronbach’s alpha: 0.786
I like my job.
Working in this nursing home ward is like being part of a large family.
This nursing home ward is a good place to work.
I am proud to work at this nursing home ward.
Morale in this nursing home ward is high.
**Working conditions** Cronbach’s alpha: 0.686
This nursing home ward does a good job of training new personnel.
All the necessary information for diagnostic and therapeutic decisions is routinely available to me.
This nursing home ward deals constructively with problem personnel.
Trainees in my discipline are adequately supervised.
The levels of staffing in this nursing home ward are sufficient to handle the number of patients.
**Stress recognition** Cronbach’s alpha: 0.694
When my workload becomes excessive, my performance is impaired.
I am less effective at work when fatigued.
I am more likely to make errors in tense or hostile situations.
Stress from personal problems adversely affects my performance.
**Perceptions of management** Cronbach’s alpha: 0.713
Senior management of this nursing home ward is doing a good job.
The management of this nursing home ward supports my daily efforts.
I am provided with adequate, timely information about events in the nursing home ward that might affect my work.

Note: Respondents rate their agreement using a five-point Likert scale: 1 = disagree strongly, 2 = disagree slightly, 3 = neutral, 4 = agree slightly, 5 = agree strongly. Reverse-coded items are indicated with*.

#### Ethical considerations

All participants received written information about the purpose of the study, and were assured that the data would be collected anonymously and treated in confidence. The study was approved by the Norwegian Social Science Data Services (Ref.no. 2016/50446)–the governmental agency for protecting survey research respondent privacy according to the Norwegian Personal Data Act [28].

### Statistical analysis

To reduce loss of cases by listwise deletion of cases with missing data, single imputation of missings were done by multiple regression analysis with SPSS v.24. Imputation by multiple regression analysis means predicting missing values of variables, using values from several other variables. For each variable with missing values imputed scores were predicted by the five answers most strongly correlated to the variable in the questionnaire [25]. Values were not imputed for those who failed to return a valid value by ticking the box “Not applicable”. We did multilevel analysis to quantify how strongly staff patient safety scores varied both across ward level and across nursing homes [29] and estimate and identify how much of the variance in the data was at the responder level and how much at organizational level [30]. Large variation at the organization level, as shown by a large intra-class correlation coefficient (ICC), would indicate that patient safety climate scores vary between individuals in a ward-specific way and that patient safety climate improvement work should be tailored to address problems of wards with low scoring respondents. An ICC of 0.10 (10%) or more is commonly seen as indicating a strong clustering of scores by organization units [31, 32]. Three empty models were estimated, one including nursing home level and respondent level, one including ward level and respondent level, and one with only the respondent level. Models were compared by the Akaike Information Criterion (AIC), where smaller values were seen as indicating better model fit.

ICC was calculated using random effects with unstructured covariance structure, using the lme4 R package and in SPSS. Confidence intervals were computed using the bootstrap.

To check whether between-ward variance could be explained by background differences among responders we applied a mixed-effects model with gender, age, years working in present ward and Norwegian as mother tongue as fixed effects and ward-level as random effect.

Within-group agreement (Rwg) (Table 3) was computed to analyze the extent to which staffs assessments were aligned within wards. Within-group agreement relates to how consistently employee perceptions in a ward are aligned, which may influence how climate measurements predict process outcomes and results [20]. Within-group agreement values above. 7 suggest strong agreement and are considered adequate to justify aggregation. The within-group agreement analysis is based on a uniform probability distribution, which makes it easier to interpret [33, 34].

Cronbach alphas were computed for the six factors and are presented in Table 1.

## Results

Of the 463 invited employees working more than 20% in the nursing homes, 288 (62.2%) responded. Response rates varied between 56.9% and 72.2% across the five nursing homes. 30% of respondents were registered nurses, 47% nursing assistants, and 16% health workers. The remaining were kitchen, laundry, secretary, and “other” staff. The average proportion of items with missing values/not applicable was 9.4%. All items of the factor model were answered by 169 respondents. After imputation of missings, 288 health care providers had responses to all items in the factor model. Table 2 shows how respondents were distributed across wards and nursing homes. Details of the respondents’ basic characteristics have been reported elsewhere [26].

**Table 2:** Distribution of respondents across wards and nursing homes.

Nursing Homes	Number of respondents	Number of wards	Median respondents per ward	Min—Max respondents per ward
1	39	3	12	2–25
2	29	3	12	5–12
3	95	12	7.5	4–14
4	70	13	7	3–8
5	55	3	12	12–31

ICC for variance at nursing home level was zero or less than one % for all factor scores. We could thus conclude that there was no nursing home factor score variation of significance.

When comparing the two-level models (ward and responder) with the single level models, the exclusion of ward level weakened model fit for four of the six patient safety climate factors, Safety climate, Job satisfaction, Working conditions and Perceptions of management as indicated by changes in AIC for these four factors, where smaller values mean better model fit. For three of these patient safety factors Safety climate, Working conditions and Perceptions of management, there was significant score variation across the wards (Table 3). The highest ICC value was for Perceptions of management at 14.2%. ICCs for Teamwork climate and Job satisfaction were 2.8% and 7.6%, respectively and insignificant. ICC for Stress recognition was zero.

**Table 3:** Total variance of the six patient safety climate factor scores, partitioned by individual and ward (i.e., ward) level.

Factor (All factors scaled 0–100) 288 respondents, 34 wards)	Teamwork climate	Safety climate	Job satisfaction	Working conditions	Stress recognition	Perceptions of management
Mean score (95% CI)	72.6 (69.9–75.3)	71.5 (68.3–74.6)	81.6 (78.8–84.5)	65.3 (61.8–68.7)	69.7 (66.9–72.5)	70.6 (66.7–74.5)
ICC: Proportion of ward level variance to total variance (95% CI)	2.76% (0.00%-10.65%)	11.60% (1.01%-23.83%)	7.61% (0.00%-17.31%)	12.81% (1.82%-24.61%)	0.00% (0.00%-7.01%)	14.07% (2.51%-25.27%)
Change in AIC value when ward level was removed from model. Smaller AIC value means better model fit.	1.2	-6.8	-2.2	-8.1	2	-10.9
Median Within-group agreement (Rwg) N = 34 wards	.77	.82	.83	.81	.62	.72
Range of Within-group agreement (Rwg) N = 34 wards	.29–1.0	.41-.98	.31-.99	.13-.98	-.07-.98	.09-.96

To check whether ward-level variation in Table 3 might reflect staff background differences from ward to ward, and not ward differences in organization culture, we included the following individual characteristics: gender, age, length of work experience at the nursing home, and Norwegian as mother tongue to the two-level model. The inclusion of sociodemographic explanatory variables into the multilevel regression analysis models improved the models’ fit to the data considerably, as shown by the large AIC-value reduction for all factors, ranging from 384.6 for the factor Perceptions of management to 287.0 for the factor Safety Climate, as shown in S1 Table. The included responder background variables eliminate ward level variation of the Safety climate factor score, but not of the Working conditions and Perceptions of management factor scores. For these two factors the ICCs even increased.

As there was no variance at nursing home level, we did not include nursing home level in this analysis.

Variation in median, mean, minimum and maximum factor scores across separate wards for each factor is presented in S1 Fig.

## Discussion

Climate score variance across wards was significant for the factors Safety climate, Working conditions, and Perceptions of management. All three factors had noticeable ICCs and sufficient between-ward heterogeneity to be considered organizational climates [19].

Control for responder sociodemographic background characteristics eliminated ward level factor score variation for Safety Climate, but not for Working conditions and Perceptions of management. For Working conditions and Perceptions of management ward level variance even increased, indicating that actual ward differences were hidden in the empty models, in which staff age, gender, mother tongue and job experience were not taken into account. The significant clustering of the factor scores Safety climate, Working conditions, and Perceptions of management indicates that leaders can address patient safety at organizational level by using climate survey scores to identify nursing home wards with high and low climate scores. This is important for clinicians and patients in wards with lower scores, where the SAQ-A may identify potentials for and perhaps lead to improvement.

In general, 42 000 people with and without dementia are residing in one of the 955 nursing homes in Norway, most of them—about 31 000—in long-term care units [35]. The average nursing home has 44 residents (range 8–200). The usual nursing home unit has 8 to 14 residents, usually in single-bed rooms (98%). Almost 90% of all institutions are owned and run by the municipalities, and more than 145 000 registered nurses and lisenced practical nurses are responsible for care and treatment. Nursing home care in Norway is a standardized service, available in all municipalities. It is paid for by the Government, which requires homogeneous provision of care for the frailest old. The included nursing homes in Tønsberg are comparable to usual nursing home standard, staffing and education level at baseline. However, individual variations for instance in connection with leader awareness and engagement are possible.

In a previous paper we already found that considerable parts of the patient safety climate factor score variations in hospitals were at ward and department levels [26]. More variation was seen at ward level than at department level. We concluded that patient safety culture improvement efforts in hospitals should not be limited to all-hospital interventions or interventions aimed at entire departments, but also include involvement at the ward level, selectively aimed at low scoring wards. In the present study, we have found that the same principle applies to nursing homes. Improvement tools to help discuss measurement results are developed and have been shown effective in dealing with ward-specific patient safety problems [7, 36].

A strength of the study is that besides studying between-ward variance, we also evaluated the climate strength by calculating within-groups agreement, thereby quantifying the degree to which patient safety is a shared concern within organizational units. It is relevant when applying the tool in practical improvement work; the stronger the climate, the more it predicts care processes and outcome [19].

The Stress recognition factor lacked variance between wards. In a previous patient safety climate study in departments of non-psychiatric care in a Norwegian hospital no between-site variation in staff perceptions of Stress recognition was found [37]. An explanation may be that the items reflect individual level attitudes and not a group phenomenon. Therefore, the factor is probably not useful for organizational climate measurements.

Staff perception of Teamwork climate and Job satisfaction were good in most wards, with little variation between wards. The variance at ward level for Teamwork climate and Job satisfaction was low and insignificant, indicating low between-ward heterogeneity. The results means that the Teamwork climate and Job satisfaction factors are less useful for identifying wards with improvement opportunities in nursing homes. This stands in contrast to a hospital study where the Teamwork climate and Job satisfaction factors produced significant between-ward variance [37]. The small ICCs produced by these two factors in nursing homes may be due to differences, e.g., in the presence or absence of medical doctors.

Hospital studies have found that medical doctors have significantly more positive Teamwork climate perceptions than do nurses [38, 39]. The difference in Teamwork climate factor and Job satisfaction factor variance between wards in hospitals and nursing homes may therefore perhaps be related to differences in the composition of professions. The extent to which doctors and nurses expect that voicing patient safety concerns will be met with respect within their team may for example have an impact [40]. That will naturally depend on interpersonal relations and support across professional boundaries to make patients safe. Positive experiences may create positive perceptions of the two factors as well as good relationships and trust between doctors and nurses. Bad experiences may, on the contrary, create distrust and negative perceptions of the same factors. In this way, measurements of Teamwork climate and Job satisfaction may expose how relationships between and within professions vary across hospital wards. In nursing homes, where differences in professional background are smaller, relationships between staff members vary less, which is reflected in little variance in the measurements.

In this study, we did not measure the nursing home care process, or its outcome. We therefore cannot validate whether variation in climate scores produces variation in care processes and outcome. This relationship has, however, been established in other studies [11, 12]. It would also have been useful to have data on leadership practices regarding dialogue meetings with staff and the extent of patient safety issues on board meeting agendas [41]. With such data we could have studied if the variation was related to leadership activities [22].

The results in this study were fed back to each of the participating nursing home wards with five or more respondents. Each ward was shown its own scores and compared with the scores of all the other participating wards, the latter presented anonymously, so no accidental reader could identify the other wards. The healthcare providers and supervisors were encouraged to focus on specific factors related to patient safety in their own wards and to discuss possible strategies for improvement.

## Conclusion

Staff perception of the Norwegian SAQ-A factor scores Safety climate, Working conditions and Perceptions of management varied significantly across wards in nursing homes. The results mean that these climate survey scores may probably be used to identify wards in nursing homes with high and low risk of adverse events. Patient safety improvement work in nursing homes should be guided by such measurements so that improvement resources are spent where they are most needed.

## Supporting information

S1 Table Ward-level variation in factor scores after inclusion of sociodemographic explanatory variables (gender, age*, years of employment at current nursing home* and Norwegian-or-other mother tongue). (DOCX)

S1 Fig Median, mean, minimum and maximum factor scores across separate wards for each factor. (DOCX)

S1 Data File (SAV)
