# Supplementary material for: Opportunities for improvement in nursing homes: Variance of six patient safety climate factor scores across nursing homes and wards—Assessed by the Safety Attitudes Questionnaire
Source: PLoS One. 2019 Jun 19;14(6):e0218244. doi: 10.1371/journal.pone.0218244 (PMC6584014; doi:10.1371/journal.pone.0218244)
Supplement: S1 Table — (DOCX) [file pone.0218244.s001.docx]

S1 Table. Ward-level variation in factor scores after inclusion of sociodemographic explanatory variables (gender, age*, years of employment at current nursing home* and Norwegian-or-other mother tongue)

| Factor | Change in AIC-value when AIC value of sociodemographic model is subtracted from AIC value of empty two-level model. Smaller AIC value means better model fit. | Residual ward level variance (ICC) of sociodemographic model (CI_95_) |
| --- | --- | --- |
| Teamwork Climate | 2286.9 - 1933.6 = 353.3 | 0 % (95% CI: 0.00% - 8.19%) |
| Safety Climate | 2012.6 - 1725.6 = 287.0 | 9.37 % (95% CI: 0.00% - 22.51%) |
| Job satisfaction | 2247.7 - 1904.6 = 343.1 | 2.75 % (95% CI: 0.00% - 13.64%) |
| Work Conditions | 2095.6 - 1797.8 = 297.8 | 13.12 % (95% CI: 1.36% - 27.49%) |
| Stress Recognition | 2367.2 - 2015.9 = 351.3 | 0.00 % (95% CI: 0.00% - 8.84%) |
| Perception of Management | 2439.7 - 2055.1 = 384.6 | 16.95 % (95% CI: 4.75% - 30.32%) |

*Categorical variables (age 5 categories, years of employment 6 categories), entered as sets of dummies
