# Supplementary material for: Opportunities for improvement in nursing homes: Variance of six patient safety climate factor scores across nursing homes and wards—Assessed by the Safety Attitudes Questionnaire
Source: PLoS One. 2019 Jun 19;14(6):e0218244. doi: 10.1371/journal.pone.0218244 (PMC6584014; doi:10.1371/journal.pone.0218244)

**S1 Figure.** **Median, mean, minimum and maximum factor scores across separate wards for each factor.**

1. Teamwork climate factor scores across separate wards


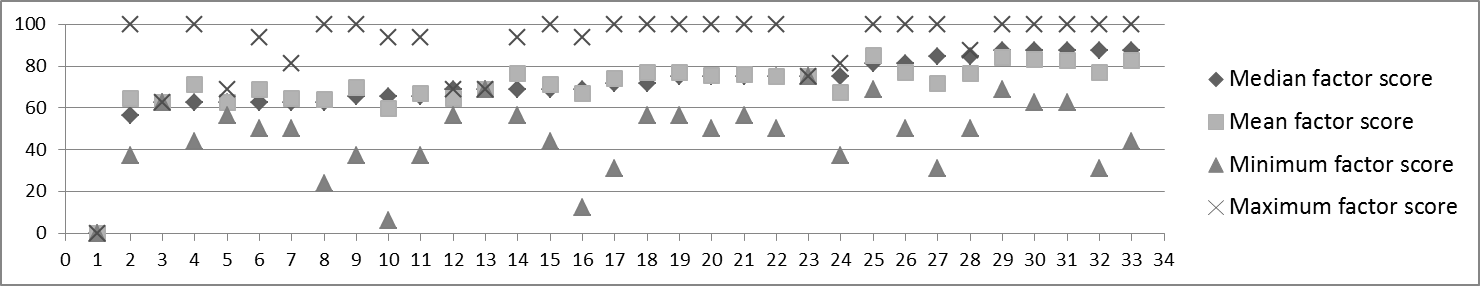


1. Safety climate factor scores across separate wards


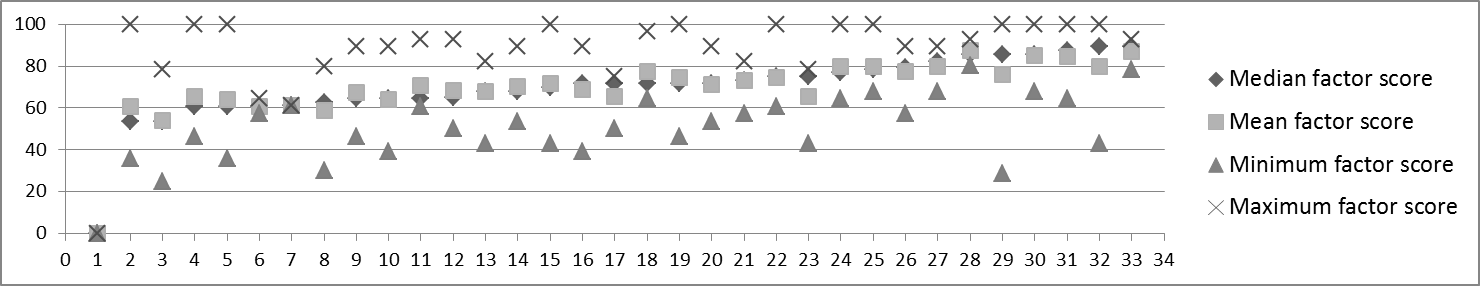


1. Job satisfaction factor scores across separate wards


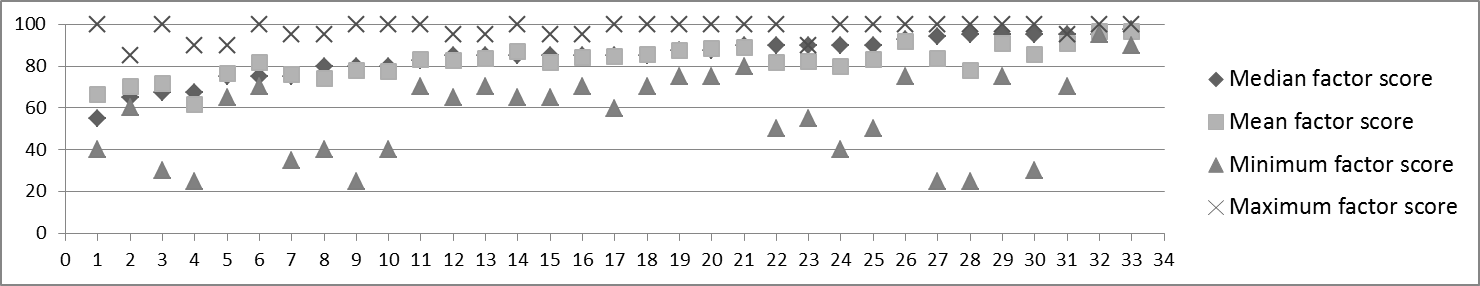


1. Working conditions factor scores across separate wards


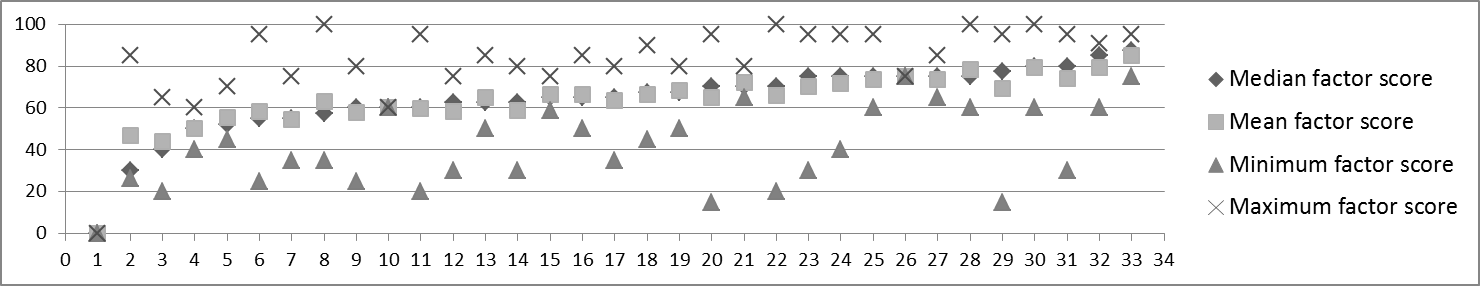


1. Stress recognition factor scores across separate wards


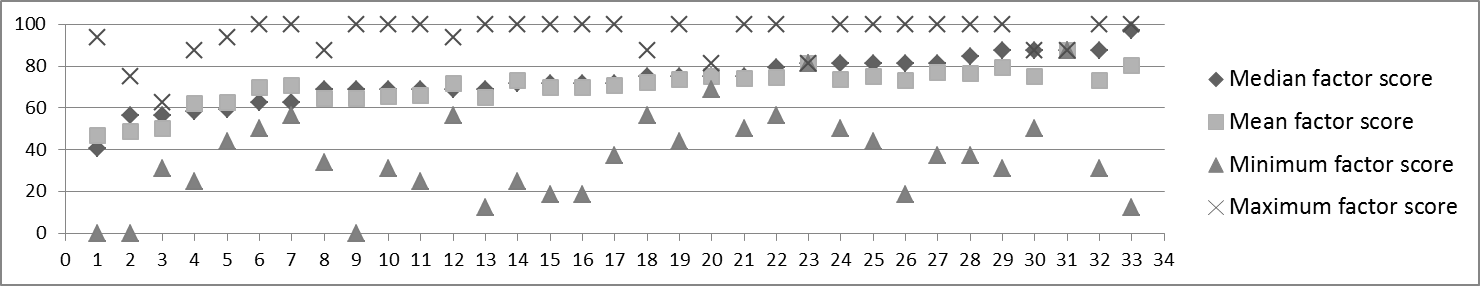


1. Perceptions of management factor scores across separate wards


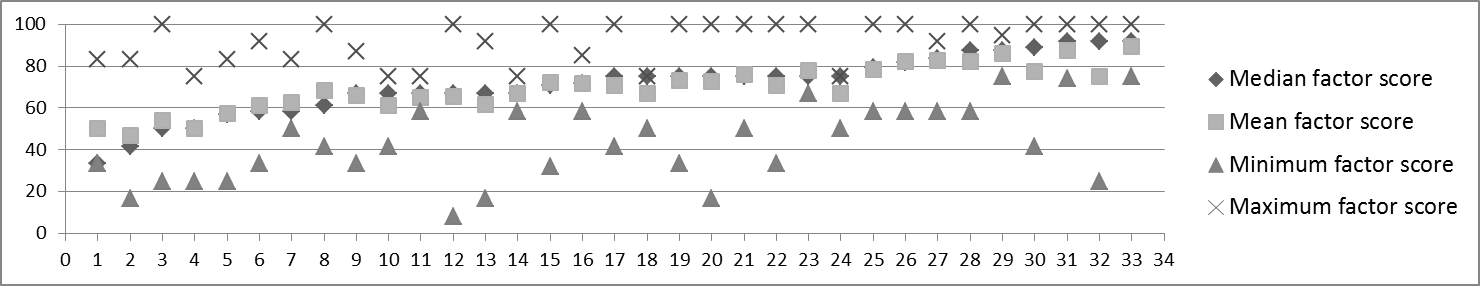

Supplement: S1 Fig — (DOCX) [file pone.0218244.s002.docx]
